# Impact of Hurricane Irene on *Vibrio vulnificus* and* Vibrio parahaemolyticus* concentrations in surface water, sediment, and cultured oysters in the Chesapeake Bay, MD, USA

**DOI:** 10.3389/fmicb.2014.00204

**Published:** 2014-05-07

**Authors:** Kristi S. Shaw, John M. Jacobs, Byron C. Crump

**Affiliations:** ^1^Horn Point Laboratory, Center for Environmental Science, University of MarylandCambridge, MD, USA; ^2^Cooperative Oxford Laboratory, National Centers for Coastal Ocean Science, National Ocean ServiceOxford, MD, USA; ^3^College of Earth, Ocean, and Atmospheric Science, Oregon State UniversityCorvallis, OR, USA

**Keywords:** aquacultured oyster, *Vibrio vulnificus*, *Vibrio parahaemolyticus*, sediment resuspension, wind event, Chesapeake Bay, estuary, storm event

## Abstract

To determine if a storm event (i.e., high winds, large volumes of precipitation) could alter concentrations of *Vibrio vulnificus* and *V. parahaemolyticus* in aquacultured oysters (*Crassostrea virginica*) and associated surface water and sediment, this study followed a sampling timeline before and after Hurricane Irene impacted the Chesapeake Bay estuary in late August 2011. Aquacultured oysters were sampled from two levels in the water column: surface (0.3 m) and near-bottom (just above the sediment). Concentrations of each *Vibrio* spp. and associated virulence genes were measured in oysters with a combination of real-time PCR and most probable number (MPN) enrichment methods, and in sediment and surface water with real-time PCR. While concentration shifts of each *Vibrio* species were apparent post-storm, statistical tests indicated no significant change in concentration for either *Vibrio* species by location (surface or near bottom oysters) or date sampled (oyster tissue, surface water, and sediment concentrations). *V. vulnificus* in oyster tissue was correlated with total suspended solids (*r* = 0.41, *P* = 0.04), and *V. vulnificus* in sediment was correlated with secchi depth (*r* = -0.93, *P* <0.01), salinity (*r* = -0.46, *P* = 0.02), tidal height (*r* = -0.45, *P* = 0.03)*,* and surface water *V. vulnificus* (*r* = 0.98, *P* <0.01). *V. parahaemolyticus* in oyster tissue did not correlate with environmental measurements, but *V. parahaemolyticus* in sediment and surface water correlated with several measurements including secchi depth [*r* = -0.48, *P* = 0.02 (sediment); *r* = -0.97, *P* <0.01 (surface water)] and tidal height [*r* = -0.96, *P* <0.01 (sediment), *r* = -0.59, *P* <0.01 (surface water)]. The concentrations of *Vibrio* spp. were higher in oysters relative to other studies (average *V. vulnificus* 4 × 10^5^ MPN g^-1^, *V. parahaemolyticus* 1 × 10^5^ MPN g^-1^), and virulence-associated genes were detected in most oyster samples. This study provides a first estimate of storm-related *Vibrio* density changes in oyster tissues, sediment, and surface water at an aquaculture facility in the Chesapeake Bay.

## INTRODUCTION

Storm events are thought to be important mechanisms for the distribution of benthic *Vibrio* populations into the water column via resuspension of sediments associated with high winds, and flushing due to large volumes of precipitation (Randa et al., 2004; Fries et al., 2008; Wetz et al., 2008; Johnson et al., 2010). Frequent storm events in the Chesapeake Bay are associated with the summer season, a time when *Vibrio vulnificus* and *V. parahaemolyticus*, autochthonous bacteria known to cause human illness, are at their highest densities in surface waters (Wright et al., 1996; Parveen et al., 2008; Jacobs et al., 2010; Johnson et al., 2012). The frequency and intensity of storm events are predicted to escalate in response to global climate change (Goldenberg et al., 2001), with increases in peak wind intensities and near-storm precipitation (Meehl et al., 2007) likely impacting mid-Atlantic areas such as the Chesapeake Bay. In the Chesapeake Bay, a shallow, partially mixed estuary prone to tidal circulation (average depth 6.5 m), storm events may be expected to increase the overall *Vibrio* density in surface waters with relatively moderate wind speed and associated wave action. Increases in post-hurricane *Vibrio* infection has been documented (e.g., Hurricane Katrina), with a resultant need for heightened clinical awareness, particularly of wound infections, following exposure to flood waters (Centers for Disease Control and Prevention [CDC], 2005). Based on the reported increases in storm-related *Vibriosis* in other areas of the United States, it is conceivable that storm-induced increases in Chesapeake Bay *Vibrio* density may be linked to future *Vibriosis* outbreaks.

According to the U.S. Environmental Protection Agency, the Chesapeake Bay is home to 25% of the total approved shellfish harvesting waters in the United States (Environmental Protection Agency [EPA], 2011). Recently, the Chesapeake Bay has become a site of interest for oyster (*Crassostrea virginica*) aquaculture production to supplement the dwindling wild harvest, both through on-bottom (submerged land) and off-bottom (water column) leases (Maryland Department of Natural Resources, Shellfish Aquaculture Program). As of January 2013, 169 aquaculture operation permit applications (~4000 acres) were submitted to Maryland Department of Natural Resources for water-column and submerged-land leases (Webster, University of Maryland Extension, personal communication), and a total of 300 submerged-land leases (~3500 acres) and 23 water-column leases (~94 acres) permitted. A small number of new aquaculture operations are in year-round production of retail oysters, with the supposition that many new operations will soon be joining their ranks.

Summer is generally considered to be a viable oyster harvest season in Maryland, but summer is also when *Vibrio* populations reach their peak in the Bay (Wright et al., 1996; Parveen et al., 2008; Jacobs et al., 2010; Johnson et al., 2012). Studies are currently being conducted to determine ways to reduce *Vibrio* concentrations in oysters (e.g., high salinity relay), but factors influencing the accumulation of high numbers or virulent strains of *Vibrio* in oysters are not completely understood (Warner and Oliver, 2008; Johnson et al., 2010; Froelich and Oliver, 2013). Thus, the harvest of oysters during seasons when surface water *Vibrio* populations are at high densities could become a pressing issue for seafood safety. If *Vibrio* density in oysters increases after storm events, shellfish managers may need to institute shellfish harvest closure periods to allow for oyster depuration or wait for suitable environmental conditions that favor a reduction in *Vibrio* concentrations, such as cooler water temperatures.

This study was conducted to test the hypothesis that a storm event, using Hurricane Irene as a proxy, generates enough wave energy to cause resuspension of sediment that would cause an increase in oyster-tissue density of *V. vulnificus* and *V. parahaemolyticus*. Oysters were tested in Taylor-style surface-water floats (Luckenbach et al., 1999) and in on-bottom cages, to determine if there was an accumulation difference based on water column position. Results from this study provide a first estimate of storm-related *Vibrio* density changes in oyster tissues, sediment and surface water at an aquaculture facility in the Chesapeake Bay.

## MATERIALS AND METHODS

### SAMPLING SITE

The study was conducted at an oyster aquaculture facility in a mesohaline tributary of the Chesapeake Bay. The oyster farm was approximately 250,000 m^2^(6 acres) with a water depth of approximately 1.2 m (4 ft) at low tide and 2.1 m (7 ft) at high tide. Sediment types at the farm ranged from predominantly sand to predominantly silt. The sampling location within the oyster farm was chosen for the predominance of silty sediment (20.4% sand: 66.6% silt: 13.0% clay; Owens, Cornwell, University of Maryland Center for Environmental Science, personal communication), which is representative of the biodeposition typically produced by oysters (Haven and Morales-Alamo, 1972). Three sampling sub-locations were selected along the outermost matrix of oyster floats, which covered approximately 1 acre, both for sediment composition and the likelihood of the area being unprotected from wind events and resultant resuspension activity. Estimates of wind speeds and resultant wave height were made using equations from Young and Verhagen (1996). Calculations of maximum bottom-sheer stress were made according to (Sanford, 1994) incorporating an approximate bottom depth of 1 m and sand grain roughness of 0.0005 m. Sand grain roughness is a measurement of characteristic bottom roughness height for use in hydrodynamic calculations. Erosion rate was calculated using the equation E (g m^-2^ h^-1^) = Mo (kg m^-2^ s^-1^ Pa^-1^) × 3600 s h^-1^ × 1000 g kg^-1^ × (τ_b_–τ_c_) (Pa), with site-specific estimates of τ_c_ = 0.025 Pa and Mo = 0.000315 kg m^-2^ s^-1^ Pa^-1^ (τ_b_: bottom-related sheer stress; τ_c_: current-related shear stress; Pascal (Pa); Mo is erosion rate constant; Sanford, Kwon, University of Maryland Center for Environmental Science, personal communication). These calculations do not acknowledge the potential for a wave-dampening effect by the large array of oyster floats tied together at the aquaculture site, although a physical oceanographer conducting experiments at the same site shares that long period waves at the bottom of the water column are damped out by perhaps as much as 50% by the floats, but not so much that resuspension would be negated (Sanford, University of Maryland, personal communication).

### ENVIRONMENTAL SAMPLE COLLECTION

Baseline surface water, oyster, and sediment samples were collected from the field location on August 26, 2011, the day before Hurricane Irene and associated storm impacts were forecast to be present along the Maryland coastline. Subsequent samples were taken at time points 1, 4, and 8 days after Hurricane Irene. All samples were collected at approximately 10:00 A.M. to approximate a uniform water and air temperature at the time of sampling due to solar irradiation.

Surface-water samples were collected at each sampling location in sterile wide mouth polypropylene 1 L bottles (Nalgene Thermo Scientific 2105-0032) following the methods described by Jacobs et al. (2009). Surface water (200 mL) was filtered through a 0.22 μm Sterivex-GP polyethersulfone filter (Millipore, Billerica, MA, USA) using a 60 mL BD luer lock syringe (BD, Franklin Lakes, NJ, USA), wrapped in Parafilm M laboratory wrapping film (Bemis Flexible Packaging, Oshkosh, WI, USA), and sealed in a labeled 7 oz Whirlpak bag (Nasco, Fort Atkinson, WI, USA). Filters were stored on ice until return to the laboratory (~1 h), where they were stored at -20°C until DNA extraction.

### PHYSICAL/CHEMICAL MEASUREMENTS

Temperature, salinity, conductivity, and dissolved oxygen were measured using a YSI Model 85 (YSI, Yellow Springs, OH, USA) at 0.3 m depth and near-bottom (~0.3 m off bottom). Secchi depth was recorded to the nearest 0.05 m. Total suspended solids (TSS) measurements were completed using 250–400 mL of surface water, filtered onto pre-weighed 47 mm glass fiber filters (Whatman GF/F, GE Healthcare Life Sciences, Piscataway, NJ, USA).

### SAMPLE SIZE

Based on standard deviations reported in Johnson et al. (2010), sample size needed was calculated for a statistical power of 0.8, significance criterion of 0.05, and preferred detection difference of 500 CFU g^-1^. Based on this calculation, three samples were required for each depth (top and bottom), per sampling period.

### OYSTER SAMPLE COLLECTION

Oyster samples (*C. virginica*) were collected from the top (*n* = 3) and bottom (*n* = 3) of the water column on each of the four sampling dates. Collected oysters [six per sample (Kaufman et al., 2003)] had shell heights (oyster hinge to opposite edge periphery) of ~ 8 cm (3.1 in). Surface water oyster samples were collected from Taylor-style floats, which remained submerged in water continuously, and bottom-water oyster samples were enclosed in 1.3 cm mesh bags deployed inside of crab pots to keep the oysters at the bottom of the water column, but out of the sediment layer. Bottom oysters, collected from identical resident oyster stock as surface oyster samples, were deployed 1 month before the commencement of this study for acclimation purposes. Collected oysters were immediately placed on ice and processed within an hour.

Crab pots consistently had a coating of top layer sediment (~1 cm) on the bottom of the pot from being deployed in the sediment. That sediment was collected at each of the three sites by filling a 50 mL Falcon sterile polypropylene conical centrifuge tube (BD Vacutainer Labware Medical 352070). Sediment samples were placed on ice, and stored frozen at -20°C.

### OYSTER PROCESSING

On each sampling date, a total of 36 oysters were examined, divided into six samples, for a total of 144 analyzed oysters over the four sampling periods. One sample of *n* = 6 oysters were collected from both the top and bottom layers at each of three sampling strata (Kaufman et al., 2003) and were homogenized following the three-tube MPN method described in the U.S. Food and Drug Administration Bacteriological Analytical Manual (BAM; DePaola and Kaysner, 2004) with slight modifications. Briefly, oysters were scrubbed, shucked with a sterile knife into a sterile blender, diluted with an equal weight of sterile phosphate-buffered-saline (Food and Drug Administration [FDA], 1998) and blended for 90 s to create a 1:1 (wt:wt) shellfish:diluent homogenate. A 1:20 dilution of oyster homogenate was made in triplicate by adding 1 mL of the 1:1 diluted homogenate to 9 mL alkaline peptone water (APW; 1% peptone, 1% NaCl, pH 8.5 ± 0.2). Additional triplicate 10-fold dilutions to 5 × 10^-7^ were prepared volumetrically by transferring 1 mL portions into 9 mL APW. Following overnight incubation at 35 ± 2°C, the top 1 mL of tubes showing growth was collected and frozen at -20°C.

### DNA EXTRACTION, DETECTION, AND QUANTIFICATION

DNA from surface water was extracted following a modified MO BIO Powersoil extraction protocol (Jacobs et al., 2009), and DNA from sediments was extracted using the standard MO BIO Powersoil extraction protocol. Extracted DNA was stored at -80°C. Quantitative PCR was used to quantify CFU mL^-1^ in water and CFU g^-1^ in sediment. The reported extraction efficiency of surface water and sediment samples using their respective methods were comparable (Jacobs et al., 2010; Lloyd et al., 2010).

DNA template was obtained from MPN cultures by producing crude cell lysates by boiling 1 mL aliquots of APW cultures in 2 mL micro-centrifuge tubes for 10 min. Following boiling, tubes were plunged into ice until cool and then centrifuged at 14,000 × *g* for 2 min. Supernatant template was added to real-time PCR reactions (3–5 uL; see PCR methods) to determine presence or absence of *V. vulnificus* and *V. parahaemolyticus* in cultured samples. Bio-rad CFX96 Touch^TM^ Real-Time PCR Detection System (Bio-rad, Hercules, CA, USA) was used to confirm the species with primers designed to detect *V. vulnificus* (Panicker and Bej, 2005) or *V. parahaemolyticus* (Nordstrom et al., 2007). Following initial detection, samples testing positive for either species were subjected to further PCR testing for virulence genes (*V. vulnificus*: virulence correlated gene, clinical variant (*vcgC*; Baker-Austin et al., 2010); *V. parahaemolyticus*: thermostable direct hemolysin (*tdh*), thermostable related hemolysin (*trh*) genes (Nordstrom et al., 2007).

Quantitative PCR was performed on surface water and sediment sample extracts by using 2.50 uL of 10X PCR Buffer (Qiagen, Valencia, CA, USA), 1.25 uL of 25 mM MgCl_2_ (Qiagen), 0.50 uL of 10 mM dNTP’s solution (Qiagen), 5 uL Q solution (Qiagen), 0.45 uL of 5 U/uL TopTaq DNA polymerase (Qiagen), 0.188 uL of 10 uM internal control primers (each), 0.375 uL of 10 uM internal control probe, 2 uL internal control DNA, 0.50 uL of 10 uM primer (each), 0.188 uL of 10 uM probe, and 3 uL DNA template per reaction, with the exception of the *V. vulnificus vcgC* assay, in which 5 uL of DNA template was used. DNase–RNase free water was added in a quantity sufficient for a 25 uL total reaction volume. Two-stage qPCR cycling parameters were optimized to the conditions as described in Shaw et al. (2014). A unique internal control, including a primer set, probe and internal control DNA, was incorporated simultaneously into each assay, excluding *V. vulnificus vcgC*, to test for the presence and influence of inhibitors (Nordstrom et al., 2007). Positive controls used for each qPCR were *V. parahaemolyticus* USFDA TX2103 and *V. vulnificus* ATCC 27562. Standard curves were constructed as reported in Jacobs et al. (2010) from spiked environmental water and used during each qPCR analysis with appropriate parameters. Cycle threshold (Ct) value was plotted against the slope of the standard curve to determine PCR unit quantity.

### MOST PROBABLE NUMBER CALCULATION USING QPCR RESULTS

Corresponding qPCR-MPN values were derived using the U.S. Food and Drug Administration MPN calculator, downloaded from the online publication “Bacteriological Analytical Manual, Appendix 2: Most Probable Number from Serial Dilutions.”^1^

### STATISTICAL ANALYSIS

Statistical analysis was completed using Intercooled Stata 9.1 for Macintosh statistical software (StataCorp LP, College Station, TX, USA). Oyster MPN g^-1^, sediment and surface water data (CFU mL^-1^) were log transformed (log_10_) to equalize variances. Each data set was analyzed for normality. Normally distributed oyster MPN g^-1^data were analyzed with multivariate analysis of variance (MANOVA) to test for differences in sampling location (top vs. bottom oyster concentrations) and sampling date for each species of *Vibrio*. Surface water and sediment samples were tested with one-way analysis of variance (ANOVA). Data sets not meeting normality criteria were analyzed with Kruskal–Wallis non-parametric rank test for differences in sampling location and sampling date. Pearson pairwise correlation analysis was conducted for the experimental variables of oyster MPN g^-1^, surface water CFU mL^-1^, sediment CFU g^-1^, MPN g^-1^, salinity, temperature, TSS, dissolved oxygen, tidal height, and secchi depth. Spearman’s rank correlation analysis was used for non-normally distributed data. Due to low sample numbers, virulence associated gene (*tdh* and *vcgC*) concentrations were not included in correlation analysis.

## RESULTS

### HURRICANE DETAILS

**FIGURE 1:**
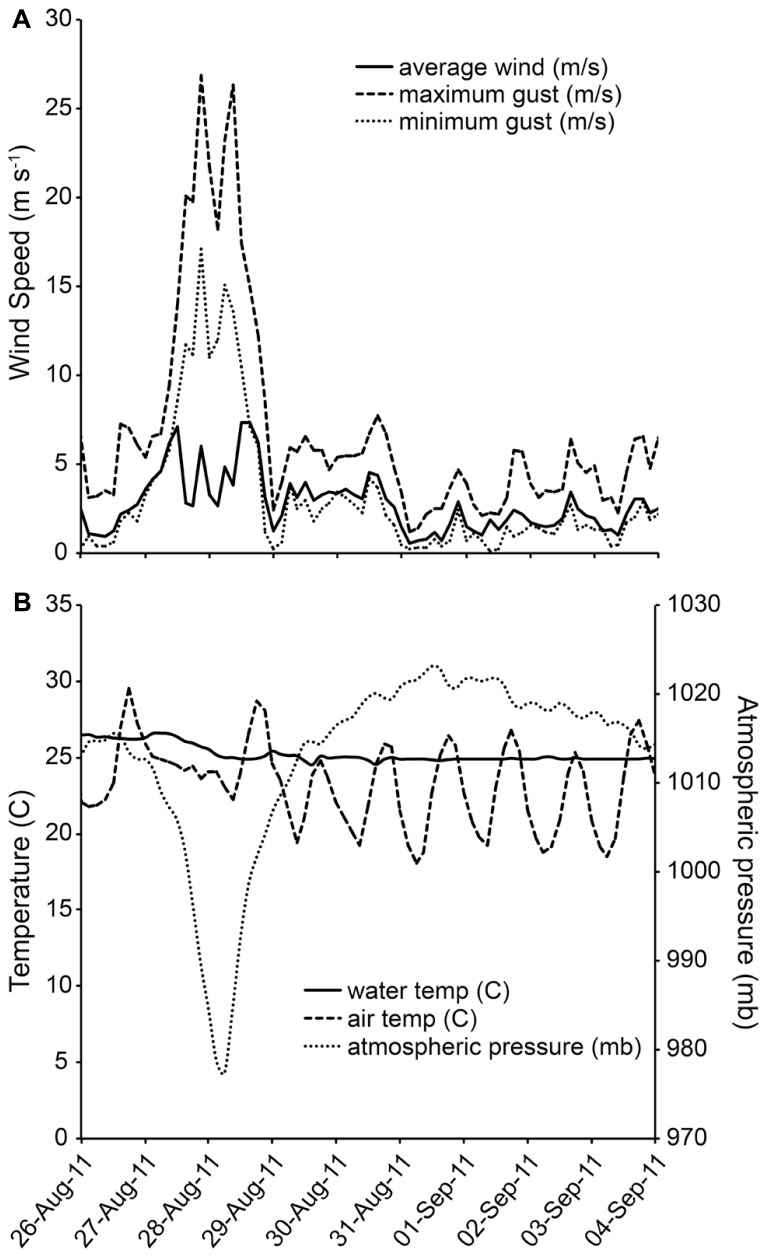
**(A,B)** Wind speed and direction at study site during Hurricane Irene Data from NOAA station CAMM2.

During the early morning hours of August 28, 2011, Hurricane Irene was just off the Delmarva coastline and the associated winds and rain impacted the Chesapeake Bay region. At the study site, there were ~18.4 cm (7.23 in) of rainfall (NOAA, 2011). Wind gusts were recorded in excess of 26 m s^-1^(58 MPH). Highest sustained winds were measured at 19.5 ms^-1^ (44 MPH) at 23:30 h on August 27, 2011 (Avila and Cangialosi, 2011; **Figure 1A**). Barometric pressure over the area reached a minimum of 976.2 mb at ~18:40 h on August 28, 2011 (**Figure 1B**). Tidal height did not deviate from the predicted normal height on the first day of sampling, so there was no hurricane-related tidal forcing at the first sampling time point.

### PHYSICAL AND CHEMICAL CONDITIONS

**FIGURE 2:**
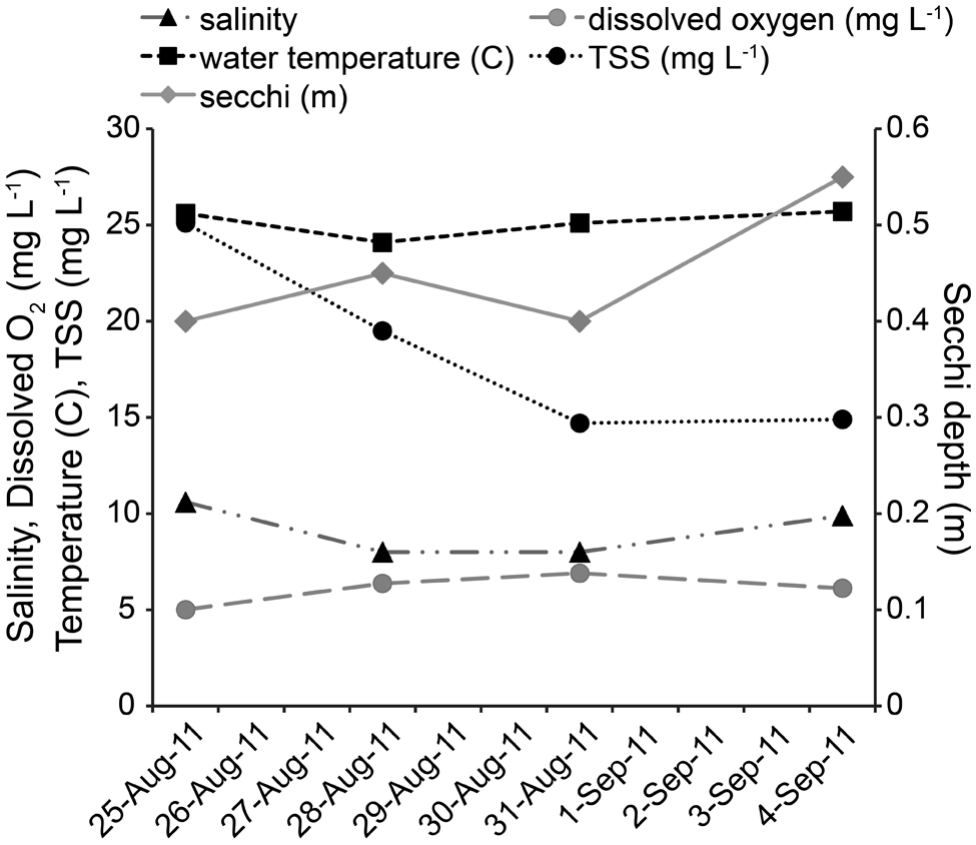
**Physical and chemical measurements of the environment**.

All physical and chemical measurements, whether taken at ~0.3 m below the surface or ~0.3 m from bottom, were found to be the same on each sampling date. As no water column stratification was detected, only one value per parameter is reported for each sampling date. Twenty-four hours after Hurricane Irene, salinity at the study site decreased from 10.6 to 8.0, and by day 8 returned to 9.9. Dissolved oxygen increased from 5.01 mg L^-1^ to 6.37 mg L^-1^ after the storm, and remained above 6 mg L^-1^. Water temperature decreased from 25.6°C to 24.1°C after the storm and by day 8 increased to 25.7°C. Secchi depth increased from 0.4 to 0.45 m on the day after the storm, returned to 0.4 m on day 4, and increased to 0.55 m on day 8 (**Figure 2**). TSS started at 25.1 mg L^-1^ and decreased over the course of the study to 19.5 mg L^-1^ (day 1), 14.7 mg L^-1^ (day 4), and 14.9 mg L^-1^ (day 8). Tidal height ranged from low tide during initial sampling efforts [pre-storm: 0.20 m above mean lower low water (MLLW), Day 1: 0.15 m above MLLW] to high tide (day 4: 0.38 m above MLLW; day 8: 0.55 m above MLLW). While changes in temperature, salinity, dissolved oxygen, secchi depth, and TSS were small, tidal height was significantly correlated with temperature (*P* = 0.001, *r* = 0.6251), TSS (*P* <0.001,* r* = -0.7512), and secchi depth (*P* <0.001, *r* = 0.6621).

### RESUSPENSION CALCULATIONS

Rates of erosion were calculated based on highest wind gusts (26.9 and 22.6 m s^-1^) and highest sustained wind speeds (9-9.8 m s^-1^). Most winds during the storm were moving in a north-northeast or northeast direction. Erosion rates were predicted to range from 2,343 to 3,616 g m^-2^ h^-1^ during periods of wind gusts and 487 to 730 g m^-2^ h^-1^ during highest sustained winds. Given the lowest wind speed (m s^-1^) during the height of the storm, the oyster farm would have expected an erosion rate of ~3 × 10^5^ g sediment h^-1^.

### Vibrio Vulnificus

#### Oyster MPN

*Vibrio vulnificus* oyster MPN g^-1^ data were not normally distributed and Kruskal–Wallis non-parametric rank test determined no statistical difference in oyster *V. vulnificus* (MPN g^-1^) by location (top vs. bottom) or by date sampled. Spearman’s rank correlation analysis of oyster *V. vulnificus* MPN g^-1^ showed significant associations with TSS (*P* = 0.0455, *r* = 0.4119; **Table 1**).

**Table 1:** Correlation table of environmental parameters and *Vibrio* concentrations in oysters, sediment and surface water.

	Oyster *Vp*	Oyster *Vv*	Surface water*Vp*	Surface water*Vv*	Sediment *Vp*	Sediment *Vv*
	log MPN g^-1^	log MPN g^-1^	log CFU mL^-1^	log CFU mL^-1^	log CFU mL^-1^	log CFU mL^-1^
Oyster *Vv*	0.2155* *P =* 0.3119					
Surface water *Vp*	-0.2258 *P =* 0.2888	-0.3562 *P =* 0.0875				
Surface water *Vv*	-0.2768 *P =* 0.1903	-0.3562 *P =* 0.0875	0.9595 *P =* 0.0000 (*n* = 24)			
Sediment *Vp*	0.1452 *P =* 0.4985	0.2338 *P =* 0.2716	0.3671 *P =* 0.0776	0.1056 *P =* 0.6235		
Sediment *Vv*	-0.2633 *P =* 0.2137	-0.3562 *P =* 0.0875	0.9866 *P =* 0.0000 (*n* = 24)	0.9882 *P =* 0.0000 (*n* = 24)	0.2113 *P =* 0.3215	
Salinity (ppt)	0.1948 *P =* 0.3616	0.2024 *P =* 0.3429	-0.4193 *P =* 0.0414 (*n* = 24)	-0.3787 *P =* 0.0680	0.0551 *P =* 0.7982	-0.4641 *P =* 0.0224 (*n* = 24)
Temperature (°C)	0.0199 *P =* 0.9266	0.0167 *P =* 0.9383	-0.3369 *P =* 0.1074	-0.1351 *P =* 0.5292	-0.5019 *P =* 0.0124 (*n* = 24)	-0.2799 *P =* 0.1853
TSS (mg L^-^^1^)	0.1616 P = 0.4507	0.4119 *P =* 0.0455	0.2811 *P* = 0.1834	0.1034 *P =* 0.6306	0.8569 *P =* 0.0000 (*n* = 24)	0.1377 *P =* 0.5210
DO (mg L^-^^1^)	-0.2205 *P =* 0.3004	-0.3395 *P =* 0.1046	0.1360 *P =* 0.5264	0.2189 *P =* 0.3042	-0.5187 *P =* 0.0094 (*n* = 24)	0.2456 *P =* 0.2473
Secchi (m)	0.1762 *P =* 0.4103	0.2435 *P =* 0.2516	-0.9727 *P =* 0.0000 (*n* = 24)	-0.9143 *P =* 0.0000 (*n* = 24)	-0.4856 *P =* 0.0161 (*n* = 24)	-0.9343 *P =* 0.0000 (*n* = 24)
Tidal height (m)	-0.0563 *P =* 0.7938	-0.2338 *P =* 0.2716	-0.5903 *P =* 0.0024 (*n* = 24)	-0.3434 *P =* 0.1005	-0.9592 *P =* 0.0000 (*n* = 24)	-0.4548 *P =* 0.0256 (*n* = 24)

*Vp*, *Vibrio parahaemolyticus*; *Vv*, *Vibrio vulnificus*; MPN, most probable number; CFU, colony forming units; ppt, parts per thousand; °C, Celsius; mg L^-1^, milligrams per liter; m, meter. *Spearman’s rank correlation used.

Although non-significant statistically, a small concentration increase in average *V. vulnificus* in oysters (MPN g^-1^) was detected between the first sampling pre-storm (August 26, 2011) and 1 day after the storm (August 29, 2011; **Table 2**). Average *V. vulnificus* decreased approximately between day 1 and day 4 post-storm, and then increased between day 4 and day 8. Despite these shifts, a very small change (1.6%) was measured in total *V. vulnificus* in oysters the entire study period.

**Table 2:** *Vibrio vulnificus* (*Vv*) and *V. parahaemolyticus* (*Vp*) concentrations.

Date	*Vv* average MPN(*n* = 6)	±SE	*Vv* top average MPN (*n* = 3)	±SE	*Vv* bottom average MPN (*n* = 3)	±SE	*Vv vcgC*^1^ average MPN (*n* = 6)	±SE	*Vv* SW CFU mL^–1^ (*n* = 3)	±SE	*Vv* sediment CFU g^–1^ (*n* = 3)	±SE
26-August-11	432,373	161,196	436,571	0	428,173	360,421	789	353	827	108	363,767	172,175
29-August-11	543,770	121,405	403,654	0	683,886	232,518	393	321	318	76	296,857	106,683
1-September-11	111,911	64,932	47,100	303	176,722	129,925	105	39	3,616	1,216	669,908	431,266
5-September-11	425,318	164,324	306,647	129,924	543,990	322,566	662	52	68	9	122,769	91,153
**Date**	***Vp* average MPN(*n* = 6)**	**±SE**	***Vp* top average MPN (*n* = 3)**	**±SE**	***Vp* bottom average MPN (*n* = 3)**	**±SE**	***Vp tdh*^2^ average MPN (*n* = 6)**	**±SE**	***Vp* SW CFU mL ^-1^ (*n* = 3)**	**±SE**	***Vp* sediment CFU g ^-1^ (*n* = 3)**	**±SE**
26-August-11	32,603	8,650	46,032	1,675	19,173	13,819	658	56	14	1	9,754	6,204
29-August-11	141,405	84,267	136,092	13,419	146,331	132,793	1,239	0	7	0.5	14,791	5,555
1-September-11	14,374	7,159	20,048	13,819	8,700	11,486	293	0	49	28	20	7
5-September-11	140,253	83,326	136,761	6,632	143,744	129,981	0	0	0	0.3	7	5

#### Surface water and sediment

One-way ANOVA analysis of sediment and surface water CFU mL^-1^ determined no statistically significant difference between dates for either sediment or surface water. Pearson’s correlation analysis of sediment *V. vulnificus* revealed significant negative relationships with the environmental variables of salinity (*P* = 0.0224, *r* = -0.4641), secchi depth (*P* <0.0001, *r* = -0.9343) and tidal height (*P* = 0.0256, *r* = -0.4548). Correlation analysis of surface water *V. vulnificus* found significant associations with sediment *V. vulnificus* concentrations (*P* <0.0001, *r* = 0.9882) and secchi depth (*P* <0.0001, *r* = -0.8917; **Table 1**).

While concentration changes detected were non-significant, average *V. vulnificus* decreased in surface waters and sediment on day 1 post-storm, increased on day 4, and decreased again to the lowest of this study’s detected *V. vulnificus* concentrations for either substrate on day 8 (**Table 2**).

#### Vibrio vulnificus virulence correlated gene

The* V. vulnificus vcgC* was detected in oysters during each of the sampling dates, but concentrations were reduced during the day 1 and 4 sampling time points (393 and 105 MPN g^-1^, respectively) relative to concentrations pre-storm (789 MPN g^-1^) and on day 8 (622 MPN g^-1^; **Table 2**). The percentage *V. vulnificus vcgC* MPN g^-1^ of overall *V. vulnificus* MPN g^-1^ was appreciably the same on all sampled dates (0.2%). *V. vulnificus vcgC* was detected in both surface and bottom sampled oysters, but not in sediment or surface waters during this study.

### Vibrio parahaemolyticus

#### Oyster MPN

Multivariate analysis of variance found no statistical difference between the sampling locations or sampling dates for *V. parahaemolyticus* MPN g^-1^values of oysters. Oyster *V. parahaemolyticus* MPN g^-1^ did not correlate significantly (Pearson’s correlation) with any of the environmental variables tested (**Table 1**).

While not significant statistically, concentration changes of average overall *V. parahaemolyticus* MPN g^-1^ increased 1 day post-storm from pre-storm concentrations and decreased 4 days post-storm, with a final increase on day 8 post-storm.

#### Surface water and sediment

One-way ANOVA analysis of difference among sampling dates for sediment and surface water CFU mL^-1^ showed no statistically significant difference between dates for either sediment or surface water. Correlation analysis of sediment *V. parahaemolyticus* CFU g^-1^ revealed significant associations with the environmental variables of temperature (*P* = 0.0124, *r* = -0.5019), TSS (*P* <0.0001, *r* = 0.8569), dissolved oxygen (*P* = 0.0094, *r* = -0.5187), secchi depth (*P* = 0.0161, *r* = -0.4856), and tidal height (*P* <0.0001, *r* = -0.9592). Correlation analysis of surface water *V. parahaemolyticus* CFU mL^-1^ found a significant negative relationship with salinity (*P* = 0.0414, *r* = -0.4193), secchi depth (*P* <0.0001, *r* = -0.9727), and tidal height (*P* = 0.0024, *r* = -0.5903). Conversely, a strong and statistically positive association was found between surface water *V. parahaemolyticus* and *V. vulnificus* CFU mL^-1^ (*P* <0.0001, *r* = 0.9595) and between surface water *V. parahaemolyticus* CFU mL^-1^ and sediment *V. vulnificus* CFU g^-1^ (*P* <0.0001, *r* = 0.9866; **Table 1**).

While not statistically significant, concentration changes of average *V. parahaemolyticus* were detected, with decreases in surface waters, but increases in sediment, 1 day after the storm. Surface water *V. parahaemolyticus* then increased on day 4 post-storm and decreased on day 8 post-storm. Conversely, sediment *V. parahaemolyticus* decreased on day 4 and decreased further on day 8 (**Table 2**).

#### Vibrio parahaemolyticus tdh/trh

The *trh* gene was not detected in any of the oyster MPN cultures, nor the sediment or surface water samples. The* tdh* gene was detected in oyster MPN cultures at all time points except on day 8. Two samples were positive for *tdh* during pre-storm sampling (average 658 MPN g^-1^), and three samples were positive post-storm (day 1, 1239 MPN g^-1^; day 8, 294 MPN g^-1^). Concentrations of *tdh* decreased over the sampling period, although overall percent *V. parahaemolyticus tdh* MPN g^-1^, when compared to total *V. parahaemolyticus* MPN g^-1^, was greatest at day 4 (2.9%). The percent of sampled oysters positive for *tdh* was lowest on day 8 [(2/6) = 33%].

## DISCUSSION

**FIGURE 3:**
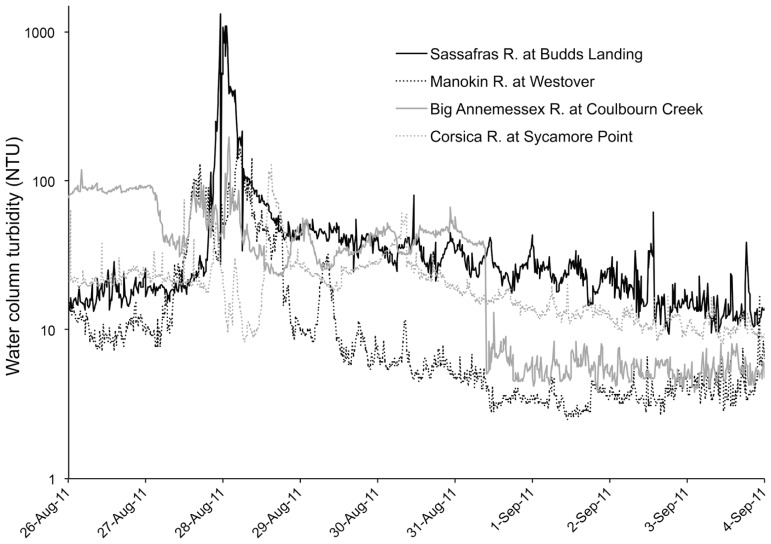
**Turbidity in Chesapeake Bay during Hurricane Irene**.

Hurricane Irene produced a significant wind event for the Chesapeake Bay region and wave action was sufficient to cause sediment resuspension at the studied aquaculture facility, according to estimates of erosion based on wind speed and direction. Additionally, there was a large amount of precipitation (18 cm) during the storm event. Although our data lacks a sampling time point during the storm, *in situ* continuous monitoring data archives of turbidity (accessed at Maryland Department of Natural Resources “Eyes on the Bay;”^2^ depict sharp spikes in nephelometric turbidity units (NTU) during the peak of the storm winds and a rapid subsequent decrease of NTU, most likely due to the large amount of rainfall experienced during the storm and a resultant flushing effect (**Figure 3**). This flushing effect may be the cause of reduced turbidity and lowered surface water CFU mL^-1^ for both *Vibrio* species 1 day after the storm.

In general, many concentrations of *V. vulnificus* and *V. parahaemolyticus* detected during this study were greater than those found in similar studies documenting the detection of these species in the same sampled matrices in the Chesapeake Bay. Maximum concentrations of *Vibrio* detected in previous studies of oyster tissue were considerably lower [*V. parahaemolyticus*: 6.0 × 10^2^ CFU g^-1^ (Parveen et al., 2008), 1.0 × 10^4^CFU g^-1^(Johnson et al., 2012); *V. vulnificus*: 1.2 × 10^4^CFU g^-1^ (Johnson et al., 2012)] than the findings of this study (*V. parahaemolyticus*: 4.1 × 10^5^ MPN g^-1^; *V. vulnificus*: 1.14 × 10^6^MPN g^-1^). In addition, Johnson et al. (2012) detected lower surface water and sediment

*V. vulnificus* concentrations [surface water: 150 CFU mL^-1^ vs. 1.2 × 10^3^ CFU mL^-1^(this study)*;* sediment: 3.5 × 10^4^ CFU g^-1^ vs. 3.6 × 10^5^ MPN g^-1^(this study)], although *V. parahaemolyticus* concentrations found in Johnson et al. (2012) were approximately double the concentrations detected in this study [surface water: 60 CFU mL^-1^ vs. 17.5 CFU mL^-1^ (this study); sediment: 1.5 × 10^4^ CFU g^-1^vs. 6.0 × 10^3^ MPN g^-1^ (this study)]. The lower oyster MPN g^-1^ and surface water/sediment *V. vulnificus* values from previous studies may be due to a difference in sampling depth for oysters (i.e., natural oyster bar depth and open water versus near shore shallows) or a difference in recovery efficiencies of methodologies used in either study, such as under-detection (culture-based methods, previous studies) or detection of non-viable cells by qPCR (direct detection, this study) in sampled surface water and sediment matrixes.

While there was large variation in the average *V. vulnificus* and *V. parahaemolyticus* cell densities in oysters, surface water, and sediment, the values quantified in each of these substrates was not significantly different over the course of the study. There was a species difference in oyster tissue concentration immediately after the storm, with *V. parahaemolyticus* increasing substantially, but *V. vulnificus* increasing only slightly. A recent, similar study (i.e., sampling frequency, salinity, and temperature range) comparing oyster, sediment, and water concentrations of *V. vulnificus* and *V. parahaemolyticus* in the Gulf of Mexico reported comparable changes in oyster tissue *Vibrio* concentrations for both species over the course of the study (Givens et al., 2014). These findings contrast with the post-storm *Vibrio* concentration changes seen in this study, suggesting a species-specific dynamic post-storm during this study. Additionally, it has been shown that *V. vulnificus* outnumbers *V. parahaemolyticus* in sediment, oyster tissue and the water column (Johnson et al., 2010). During this study, *V. parahaemolyticus* cell g^-1^ was approximately 5% of the total *V. vulnificus* cell g^-1^ in sediment, which is consistent with the findings of Johnson et al. (2010). However, despite the relative dominance of *V. vulnificus* in sediments, post-storm increases in *Vibrio* were dominated by *V. parahaemolyticus,* suggesting species-specific variation during this study in the degree to which these bacteria were resuspended from sediments or were retained in oyster tissues, perhaps differing from *V. vulnificus* in properties of adhesion to marine aggregates, which may have been subsequently filtered by oysters.

Interestingly, on day 4 post-storm, oyster tissue *Vibrio* MPN g^-1^decreased precipitously from pre-storm concentrations (-74%, *V. vulnificus*; -56% *V. parahaemolyticus*), while surface water CFU mL^-1^ and sediment CFU g^-1^increased substantially (+337 and +84%, respectively; **Table 2**). On day 8, oyster tissue *V. vulnificus* concentrations returned to pre-storm concentrations (-1.6%), while *V. parahaemolyticus* MPN g^-1^ concentrations approximately quadrupled. Conversely, surface water and sediment concentrations decreased to a fraction of their original concentrations at day 8 post-storm (-92, -66% *V. vulnificus*, respectively; -100% for both sediment and surface water, *V. parahaemolyticus*). One possible explanation for these changes is a bacterial response to the flushing effect from the wind and rain at the study site, but more likely is storm-induced changes in oyster filtration rates over the course of this study. In Givens et al. (2014), changes in *Vibrio* concentration were seen to be approximately replicated in surface waters and oyster tissues, suggesting that the opposing patterns of oyster and water *Vibrio* concentration detected in the days following Hurricane Irene were atypical.

Oysters have been shown to reduce or halt filtration during periods of high suspended solids, recommencing filtration at a normal or increased rate when water clarity returns to ambient conditions (Loosanoff and Tommers, 1948). If filtration stalled during the height of the storm and then resumed after sediment resuspension ceased, it may have explained the concomitant decrease in oyster *Vibrio* concentrations by 5–10 times (**Table 2**), while surface water *Vibrio* concentrations increased by 7–11 times on the fourth day post-Hurricane Irene (**Table 2**; **Figure 2**). However, filtration rates were not directly measured in this study and other factors, such as population turnover and physical transport, cannot be excluded as potentially important mechanisms for changes in *Vibrio* concentrations. Similar to Fries et al. (2008), who noted an increase in sediment concentrations of total *Vibrio* when Hurricane Ophelia impacted the Neuse River Estuary, NC, USA; there was also an increase in the sediment concentrations of both *Vibrio* species during the first four days post-storm (**Table 2**). However, this pattern then reversed with an overall decrease in sediment CFU g^-1^(-100%, *V. parahaemolyticus;* -66%, *V. vulnificus*). Whether this was due to a change in oyster filtration or a difference in how each *Vibrio* species was introduced into the water column as a function of resuspension, and associated particle adhesion, remains to be understood. In contrast to other studies (Fries et al., 2008; Hsieh et al., 2008; Wetz et al., 2008; Johnson et al., 2010), surface water CFU mL^-1^ decreased following the storm (**Table 2**).

Notably, virulence-associated genes of *V. vulnificus and V. parahaemolyticus* were not detected in surface waters or sediment during the course of this study, possibly due to limitations of the direct extraction method (sediment, water) in relation to the MPN enrichment method (oyster samples). This is counter to other study findings, such as Johnson et al. (2010), which reported virulence-associated *V. parahaemolyticus* genes at similar frequencies in sediment, surface water and oysters. The *V. vulnificus vcgC* gene was found routinely in oyster tissues, but the percentage of *V. vulnificus* carrying *vcgC* was elevated at the beginning and end of the study (0.2%), and reduced one day after the storm and on day 4 (0.09%). Similarly, the percentage of *V. parahaemolyticus* carrying the *tdh* virulence-associated gene was elevated before the storm and on day 4 (2%) and reduced one day after the storm (0.7%). Incidence and concentration of virulent *V. parahaemolyticus* was at its lowest point at day 8 (0%). These findings are in contrast to previous, laboratory-based studies, examining the relationship between *V. vulnificus’* virulence associated genes in oysters. These previous studies found no change in *V. vulnificus* virulence associated genes during the passage through the oyster (Groubert and Oliver, 1994; Staley et al., 2011). It is possible that the changes in virulence-associated genes percentages in this study are associated with population turnover within the oyster during the storm period.

Movement towards increased aquaculture production of oysters in the Chesapeake Bay, in combination with forecasted environmental responses to global climate change (e.g., warmer surface waters, increased frequency and/or intensity of storm events), may create a situation of higher *Vibrio* density in oysters, especially during the summer harvest season. An inventory of the last decade of tropical storms (2001–2011)^3^ in the Chesapeake Bay elucidates that at least one tropical storm or depression is routinely seen in the region each year, and at least one hurricane within each decade, with an anticipated increase in tropical weather influenced by climate change conditions. Further research is needed to determine if patterns of adherence to oyster tissues is different between *V. parahaemolyticus* and *V. vulnificus*, as well as among virulent subsets of each species. As the storm event in this study consisted of both high winds and large amounts of precipitation, it would be useful to examine storm events with a range of wind speeds and precipitation to account for the individual response variables of resuspension and surface water flushing. Additionally, the role of nutrient introduction from terrestrial sources and the impact of plankton dynamics on *Vibrio* populations should be investigated in future studies to elucidate the impact of either variable on *Vibrio* concentration in the measured substrates. Such information would help managers of shellfish harvest decide if there should be a cessation or modification (e.g., post-harvest treatment) of harvest post-storm, what winds or rainfall would be significant for a given aquaculture site, and how long that suspension or modification of harvest should be recommended.

## Conflict of Interest Statement

The authors declare that the research was conducted in the absence of any commercial or financial relationships that could be construed as a potential conflict of interest.

## Footnotes

1 http://www.fda.gov/Food/scienceResearch/LaboratoryMethods/BacteriologicalAnalyticalManualBAM/ucm109656.htm

2 http://mddnr.chesapeakebay.net/eyesonthebay/index.cfm

3 http://www.weather.gov/lwx/tropical
